# Translating conventional wisdom on chicken comb color into automated monitoring of disease-infected chicken using chromaticity-based machine learning models

**DOI:** 10.3389/fvets.2023.1174700

**Published:** 2023-06-21

**Authors:** Mohd Anif A. A. Bakar, Pin Jern Ker, Shirley G. H. Tang, Mohd Zafri Baharuddin, Hui Jing Lee, Abdul Rahman Omar

**Affiliations:** ^1^Department of Electrical and Electronics Engineering, College of Engineering, Institute of Sustainable Energy, Universiti Tenaga Nasional, Kajang, Malaysia; ^2^Center for Toxicology and Health Risk Studies (CORE), Faculty of Health Sciences, Universiti Kebangsaan Malaysia, Kuala Lumpur, Malaysia; ^3^Department of Electrical and Electronics Engineering, College of Engineering, Institute of Power Engineering, Universiti Tenaga Nasional, Kajang, Malaysia; ^4^Department of Veterinary Pathology and Microbiology, Faculty of Veterinary, Universiti Putra Malaysia, Serdang, Malaysia; ^5^Institute of Bioscience, Universiti Putra Malaysia, Serdang, Malaysia

**Keywords:** machine learning, classification model, chromaticity, agriculture, chicken comb, image processing, diseases-infected chicken, energy

## Abstract

Bacteria- or virus-infected chicken is conventionally detected by manual observation and confirmed by a laboratory test, which may lead to late detection, significant economic loss, and threaten human health. This paper reports on the development of an innovative technique to detect bacteria- or virus-infected chickens based on the optical chromaticity of the chicken comb. The chromaticity of the infected and healthy chicken comb was extracted and analyzed with International Commission on Illumination (CIE) XYZ color space. Logistic Regression, Support Vector Machines (SVMs), K-Nearest Neighbors (KNN), and Decision Trees have been developed to detect infected chickens using the chromaticity data. Based on the X and Z chromaticity data from the chromaticity analysis, the color of the infected chicken’s comb converged from red to green and yellow to blue. The development of the algorithms shows that Logistic Regression, SVM with Linear and Polynomial kernels performed the best with 95% accuracy, followed by SVM-RBF kernel, and KNN with 93% accuracy, Decision Tree with 90% accuracy, and lastly, SVM-Sigmoidal kernel with 83% accuracy. The iteration of the probability threshold parameter for Logistic Regression models has shown that the model can detect all infected chickens with 100% sensitivity and 95% accuracy at the probability threshold of 0.54. These works have shown that, despite using only the optical chromaticity of the chicken comb as the input data, the developed models (95% accuracy) have performed exceptionally well, compared to other reported results (99.469% accuracy) which utilize more sophisticated input data such as morphological and mobility features. This work has demonstrated a new feature for bacteria- or virus-infected chicken detection and contributes to the development of modern technology in agriculture applications.

## 1. Introduction

The increase in human population has forced poultry meat production to increase (1). However, mass production in the poultry industry may be more vulnerable to disease outbreaks in farmed animals due to the increased number of animals per area and prolonged usage of antibiotics (2). The World Bank reported a direct cost of $20 billion for disease outbreak events between 1988 to 2006 (3), including public and animal health costs, compensation, production, and revenue costs. Plus, indirect losses, including animal product chain, trade, and tourism, were estimated to be more than $200 billion worldwide (3). For instance, the United States and China’s poultry industry recorded huge economic losses and threats to human health due to several poultry-related diseases such as the H7N9 avian influenza virus outbreak in 2013 (4), multistate foodborne outbreak of *Salmonella* Typhimurium (5), avian influenza outbreaks in 2022 (6, 7), foodborne pathogens such as *Campylobacter*, *Escherichia coli*, *Salmonella*, and Norovirus (8), severe respiratory illness among poultry slaughter plant workers due to *Chlamydia psittaci* (9), and human infection with the influenza A (H5N6) virus of avian origin (10). Although the viruses are preventable, curable, and controllable, there is still a continuous threat that they could start a pandemic if the viruses develop the ability to spread among humans effectively. Therefore, early detection of diseases in poultry production is a primary concern to prevent a major outbreak that would affect the economy and human health.

Numerous disease detection methods have been proposed, developed, and widely applied to give early detection to prevent this catastrophe. The conventional method of detecting infected chicken was using physical examination and laboratory tests. The physical examination is a way of seeing infected chicken through observation of clinical signs or changes in behavior and physical appearance of the chicken individually. The suspected chicken will be evicted from the flocks and undergo laboratory tests such as culture (11–13), polymerase chain reaction (PCR) (14, 15), enzyme-linked immunosorbent assay (ELISA) (16, 17) and lateral flow assay (LFA) (18, 19). Biological samples such as blood, cloacal swabs, organs, and feces were collected from the suspected chicken for the test. Apart from the requirement of trained personnel to conduct the tests, these methods are considered costly due to the equipment needed, such as a thermocycler, ELISA reader, PCR buffer, syringe, swab kit, and petri dish for sampling and detecting the pathogen (20). Overall, these methods can detect infected chickens with high precision and specificity. However, many other factors, such as cost and time taken for detection, were compromised, which makes it almost impossible to be implemented, especially for large-scale poultry producers.

The rapid development of modern technology has introduced the development of biosensors to detect infections with consideration of other factors such as sensitivity, cost, efficiency, and time taken for detection (21–23). Although biosensors can detect infected chickens faster than laboratory tests with good sensitivity and accuracy, each method was considered intrusive due to the biological sample needed for the test. Non-intrusive and non-invasive techniques in giving an early warning for detecting infected chickens based on their vocalization, video, and image have been introduced with the aid of advanced information technologies, especially machine learning. Several researchers have successfully detected infected chickens based on their abnormal sounds like rales, sneezing, and coughing (24–26). However, it was challenging to detect infected chickens individually based on vocalization because more than one chicken may sneeze or cough simultaneously. Computer vision, like digital images and video, can detect and classify infected chickens in real-time, and many different methods have been proposed (27–31). However, these works carried out the classification based on locomotor and mobility of the chicken (27), differences in morphological features (28), differences in posture and feather images (29), using an abnormal swelling image (30), and the correlation of the optical flow parameters with the occurrence of hockburn in chicken (31).

In conventional understanding, the infected chicken can be detected based on the biological change in the appearance of the chicken itself, especially its comb. For example, the Newcastle disease infection would show clinical signs such as swollen comb (32), nodular lesions on its comb characterized by fowl pox disease infection (33), and fatty liver hemorrhagic syndrome would show clinical signs of a pale comb (34). Previous studies have reported on the relationship between comb color and size with the immunity system of birds using spectrophotometry (35, 36). However, these results were based upon data from red grouse (bird) combs and it is still unclear on the correlation between the comb’s chromaticity and bacteria- or virus-infection, since these works were investigating only the immunity system of the birds. To the best of our knowledge, there is no specific research work that correlates the optical chromaticity of the chicken comb with infectious diseases using image processing. Therefore, this work investigates on the effectiveness of utilizing image processing techniques incorporated with machine learning algorithms to correlate the color of the chicken comb with bacteria- or virus-infected chicken. The difference between infected and healthy chicken comb is analyzed based on chromaticity data. Since computer is a low-cost, non-invasive and non-intrusive method for detecting infected chicken, digital image colorimetry was adopted in this work. Using the chromaticity data, machine learning algorithms such as Logistic Regression, Support Vector Machine (SVM), K-Nearest Neighbors, and Decision Tree, were developed to classify the infected and healthy chickens. Each model’s performance, advantages, and disadvantages for this current application were analyzed in this study.

## 2. Image processing and machine learning algorithms

**Figure 1:**
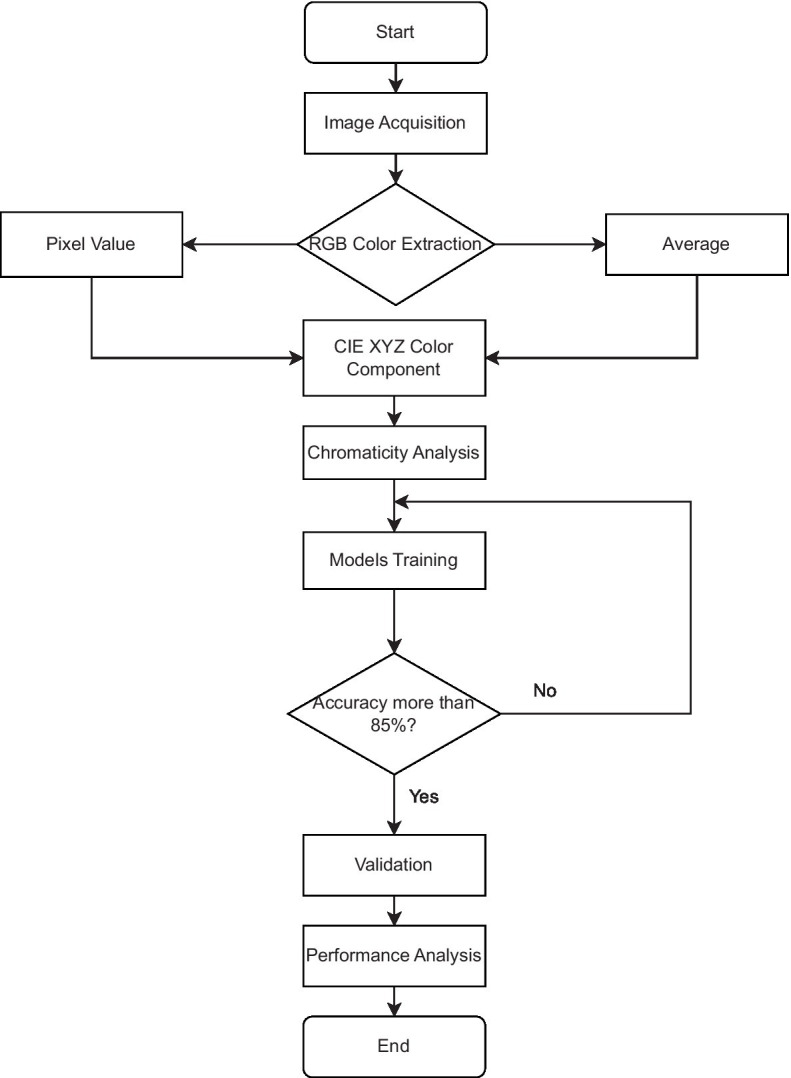
Workflow of the RGB color data extraction methods, data analysis and development of machine learning models.

A digital image is a combination of color space data, and many researchers had performed colorimetry studies based on digital image color space data for a few applications and areas (37–41). Since digital image colorimetry is a well-known method for describing perceived color, this technique was used to extract the color component of the chicken comb at pixel level and the average pixel color component bounded on the comb area. The Red Green Blue triplets, RGB values were extracted, normalized, and linearly transformed into CIE XYZ color space using the developed Python program and ImageJ software. Normalized CIE XYZ, named the CIE *xyz* component, was studied and analyzed incorporated with the machine learning model, Logistic Regression. The supervised machine learning classification algorithms, Logistic Regression, SVM with different types of kernels, KNN, and Decision Tree model were used to classify the chicken health based on the color component. The models were trained and validated to analyze the performance parameter in this current application. Figure 1 shows the workflow of this study, from the RGB color data extraction methods to the chromaticity data analysis and the development of machine learning models to classify chicken health. The details for the major stage of the method, which are image acquisition, data organization, image processing and data labeling, CIE XYZ color space, supervised machine learning algorithms, and performance parameter, are discussed in the following subsections.

### 2.1. Image acquisition

**Figure 2:**
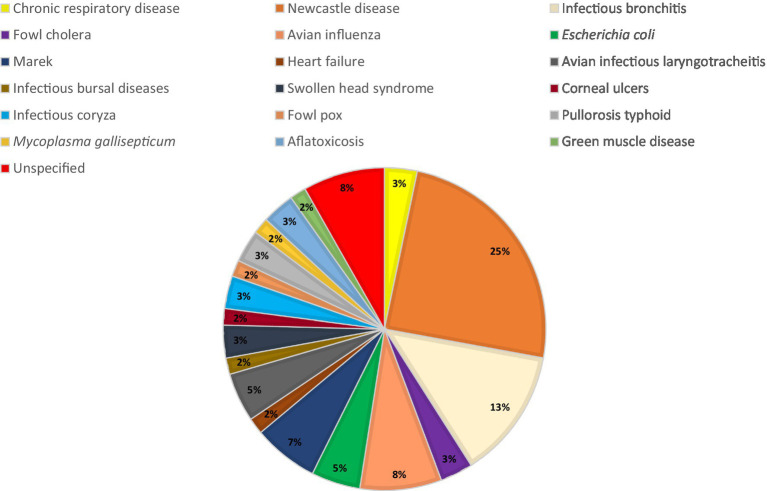
Percentage distribution of different diseases for the infected chicken image dataset.

Digital image data were manually collected from various sources such as journals, short communications, articles, veterinary websites, and blogs through the open-source Google Search engine because no specified image dataset related to this work could be obtained. A total of 122 images were downloaded and classified into two groups, healthy and infected chickens, with 61 images in each group without considering any specific quality such as resolution, lighting condition, the pixel value of the image, the distance between the camera and the chicken, and the angle view of the chicken. The images were labeled as healthy and infected based on the source’s justification. All the image data including masked chicken comb images and sources have been uploaded to a GitHub repository.^1^ All chickens were assumed to be alive based on general observation. Images were selected based on the feather color to indicate a type of chicken, and the current work considered chickens with white feathers only. However, the chicken husbandry care such as the diet, age, temperature, humidity of surroundings, and severity of the diseases were not considered in this work. As presented in Figure 2, most of the chickens in the infected class dataset were infected with Newcastle disease (25%), followed by infectious bronchitis (10%) and avian influenza (8%).

### 2.2. Data organization

The image data was split into training and validation sets to reduce bias in training the model. Eighty images were randomly picked as a training dataset for fitting the models, and the remaining images were used as the validation set. The models were validated by 42 healthy and infected chickens, which were randomly distributed but properly structured to represent all diseases. For infected chicken with a total of only 2 or 3 images, such as chronic respiratory diseases, fowl cholera, infectious coryza, swollen head syndrome, aflatoxicosis, *E. coli*, avian infectious laryngotracheitis, and pullorosis typhoid, one image was randomly picked from each group for validation. Two photos were selected for validation from each disease group containing 4 to 8 images representing Marek, avian influenza, infectious bronchitis, and unspecified diseases. The most considerable portion of the validation dataset belongs to Newcastle disease, with 23.81% (5 images out of 21 total) due to overall image acquisition. However, infectious bursal disease, *Mycoplasma gallisepticum*, heart failure, fowlpox, corneal ulcers, and green muscle disease were not included in the validation dataset, due to a lack of image data. Overall, the training dataset consists of 40 healthy and 40 infected chicken images, while the validation set consists of 21 healthy and 21 infected chicken images.

### 2.3. Image processing and data labeling

**Figure 3:**
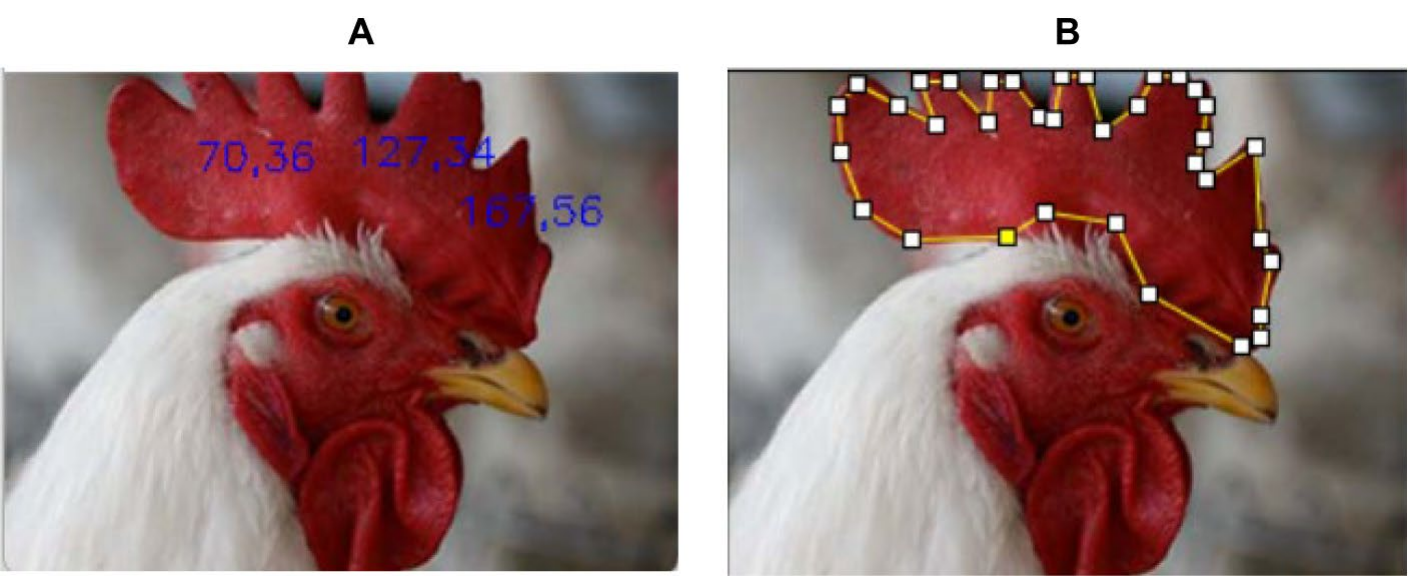
Data extraction using **(A)** pixel-level method and **(B)** pixel-averaging method.

The raw image data were not uniform in size and resolution. The image of the chicken head was cropped manually to analyze its comb color within the comb area excluding the region that has overlayed text. This work used two methods to extract the RGB value of the chicken comb. The first method was by extracting 3 RGB sample points within the area of the chicken comb, as shown in Figure 3A. The second method was by extracting the average RGB value of all pixels within the chicken comb, as shown in Figure 3B. Throughout this paper, the first method will be named the pixel-level method, and the second method will be named the pixel-averaging method.

Figure 3A shows that three sample points were taken from the image at coordinates (70.36), (127.34), and (167.56). The image coordinate was specified based on the chicken comb using the convention of width and height. Figure 3B shows that the chicken comb was manually selected to calculate the average value of all the extracted RGB values within the selected region. The RGB data was normalized and transformed into CIE XYZ color space which was discussed theoretically in the next subsection. The collected RGB and CIE XYZ color space data were saved in Macintosh (.csv) format for further analysis. The infected chicken was labeled as 0 for the true positive event, and the healthy chicken was labeled as 1 for the true negative event as described in the literature (42).

### 2.4. CIE XYZ color space

In this work, the CIE XYZ color space (43) was utilized to analyze the chromaticity of the infected and healthy chicken combs. The extracted RGB data were normalized and converted to CIE XYZ color space using the linear matrix transformation as shown in Equation 1. The formula was directly adopted from (43) the Rec. 709 RGB standards with its reference D65 white point for all images.


(1) $$\left[\begin{array}{c} X\\ Y\\ Z \end{array}\right]=\left[\begin{array}{ccc} 0.4124564 & 0.3575761 & 0.1804375\\ 0.2126729 & 0.7151522 & 0.0721750\\ 0.0193339 & 0.1191920 & 0.9503041 \end{array}\right]\left[\begin{array}{c} R\\ G\\ B \end{array}\right]$$

The XYZ values were normalized to restrict the range from 0 to 1 and denoted as *x*, *y*, and *z* values. The formulas used in normalizing the value were expressed in Equations (2)–(4).


(2) $$\begin{array}{c} x=\frac{X}{X+Y+Z} \end{array}$$

(3) $$\begin{array}{c} y=\frac{Y}{X+Y+Z} \end{array}$$

(4) $$\begin{array}{c} z=\frac{Z}{X+Y+Z} \end{array}$$

The scatter plots of *xy*, *yz*, and *xz* values were analyzed to determine the differences between the healthy and infected chickens.

### 2.5. Supervised machine learning algorithms

This research work utilized four different classifier algorithms, namely Logistic Regression, SVM, KNN, and Decision Tree. Scikit Learn library was used for pre-processing the data and training the models as specified in the package (44). The *x* and *y* chromaticity data were utilized as the features for the classifier. The chromaticity data features were standardized using the StandardScaler module from the Scikit library for faster convergence and better results. Further fundamental, theoretical, and mathematical theories of these models were well discussed in the library documentation. Hyperparameters of each model were adjusted and the best model was selected and discussed based on the confusion matrix, which was discussed in the performance parameter subsection. The advantages and disadvantages of deploying each model were also addressed for this current application in section 3.2.

#### 2.5.1. Logistic regression

The logistic regression model is a supervised machine learning model to predict the class probability, which ranges from 0 to 1 in our application. The model predicts 0 for probability ranging from 0 to 0.5, and the class belongs to the positive event or infected chicken. The theory of the logistic regression model was explained in literature (45). The logistic function was used to restrict the linear regression model’s output to a range from 0 to 1. The general logistic equation is given in Equation 5. Note that, p(y) is the function for the probability value, and variable y in the equation corresponds to the input function for the logistic equation.


(5) $$\begin{array}{c} p\left(y\right)=\frac{1}{1+e^{-y}} \end{array}$$

Since the logistic regression was restricting the linear regression model, the final equation for the model is stated in Equation 6.


(6) $$\begin{array}{c} p\left(f\left(x_{1},x_{2}\right)\right)=\frac{1}{1+e^{-\left(B_{0}+B_{1}x_{1}+B_{2}x_{2}\right)}} \end{array}$$

where $f\left(x_{1},x_{2}\right)$ is the sigmoid input function for the logistic equation, *x_1_* and *x_2_* correspond to the predictor or chromaticity data for the classifier, and *B_0_*, *B_1_*, and *B_2_* correspond to the coefficient of the predictors. Current work will utilize the sigmoid input function $f\left(x_{1},x_{2}\right)$ , to analyze and correlate the chromaticity data and the health status of the chickens. The general function is stated in Equation 7.


(7) $$\begin{array}{c} f\left(x_{1},x_{2}\right)=B_{0}+B_{1}x_{1}+B_{2}x_{2} \end{array}$$

The iteration of the cost function, C parameter, was carried out and the highest accuracy performance was analyzed.

#### 2.5.2. Support vector machine

SVMs are a popular supervised learning technique for outliers’ detection, regression, and classification. SVM algorithms take data as input and transform it into the desired form using a set of mathematical functions referred to as the kernel. Given that the ScikitLearn library offers four distinct kernel functions (44)—Linear, Polynomial, Radial Basis Function (RBF), and Sigmoid—the current work will develop the models across all four kernels. The Linear, Polynomial, RBF, and Sigmoid kernel functions are given in Equations (8)–(11), respectively.


(8) $$\begin{array}{c} K\left(x_{1},x_{2}\right)=x_{1}.x_{2}\; \end{array}$$

(9) $$\begin{array}{c} K\left(x_{1},x_{2}\right)={\left(\gamma x_{1}.x_{2}+r\right)}^{d} \end{array}$$

(10) $$\begin{array}{c} K\left(x_{1},x_{2}\right)=e^{-\gamma {\left|x_{1}-x_{2}\right|}^{2}} \end{array}$$

(11) $$\begin{array}{c} K\left(x_{1},x_{2}\right)=\tanh\left(\gamma x_{1}.x_{2}+r\right) \end{array}$$

where $x_{1}$ and $x_{2}$ are chromaticity data features in vectors form, d is the degree, γ is gamma, and r is the parameter of the kernel projection. Hyperparameters for tuning each model, were iterated, and the model that produced the best accuracy performance were selected and compared.

#### 2.5.3. K-nearest neighbor

KNN algorithm is a non-parametric classifier that uses positional information to categorize or forecast how a single data point will be grouped. The general matric for calculating the distance between data points is Minkowski and for the current application, we used the Euclidean distance formula. The general equation is stated in Equation 12.


(12) $$\begin{array}{c} d\left(i,j\right)=\sqrt{{\left|x_{i1}-x_{j1}\right|}^{2}+{\left|x_{i2}-x_{j2}\right|}^{2}} \end{array}$$

where d(i,j) is the function for calculating the distance between training point i and data point j . $x_{i1}$ and $x_{i2}$ are the chromaticity data of the training set, while $x_{j1}$ and $x_{j2}$ correspond to the chromaticity data of the predictor or validation data.

For model training purpose, the k-value represents the number of closest neighbors and is the primary hyperparameter value for KNN. Since the k-value needed to be established appropriately (46), the value was iterated from 1 to 20, and the k-value with the best performance was discussed.

#### 2.5.4. Decision tree

Decision Tree is a non-parametric supervised learning method for classification and regression to create a model that predicts the value or class of a target variable by learning simple decision rules concluded from the data features. The library provided two criteria settings, “Gini” and “Entropy,” to measure the quality of the split in decision rules. The corresponding formulas are stated in Equations (13) and (14).


(13) $$\begin{array}{c} Gini\left(D\right)=1-\overset{k}{\underset{i=1}{\sum }}p_{i}^{2} \end{array}$$

(14) $$\begin{array}{c} Entrophy\left(D\right)=\overset{k}{\underset{i=1}{\sum }}-p_{i}^{2}\log_{2}\left(p_{i}\right) \end{array}$$

D corresponds to the dataset, k is the number of classes in the dataset, and $p_{i}$ is the ratio of the class. Both “Gini” and “Entropy” as provided in the library were utilized for the criterion setting to measure the quality of the split, and the best model was chosen for further analysis and comparison.

### 2.6. Performance parameter

The model’s performance was analyzed using the confusion matrix method based on five parameters: sensitivity, specificity, precision, negative predictive value (NPV), and accuracy (42). The performance of the classification model was evaluated based on the convention stated in the literature. Seven models were trained and validated: Logistic Regression, SVM with Linear, Polynomial, RBF and Sigmoid kernels, KNN, and Decision Tree. The performance of each model was investigated, compared, and analyzed. The implementation of the models in practical applications was also discussed in the present study based on the current application.

## 3. Results and discussion

This section was organized according to three main subsections; chromaticity analysis, supervised machine learning results, and comparison with other related works. The first phase of analysis revealed the impact of infection on the chromaticity of the chicken comb, and the correlation between chromaticity and health status is discussed. Next, the performance of each developed model is discussed, analyzed, and compared accordingly. Lastly, the performances of all the models are comprehensively compared with reported machine-learning algorithms related to this current application for classifying infected chickens.

### 3.1. Chromaticity analysis

**Figure 4:**
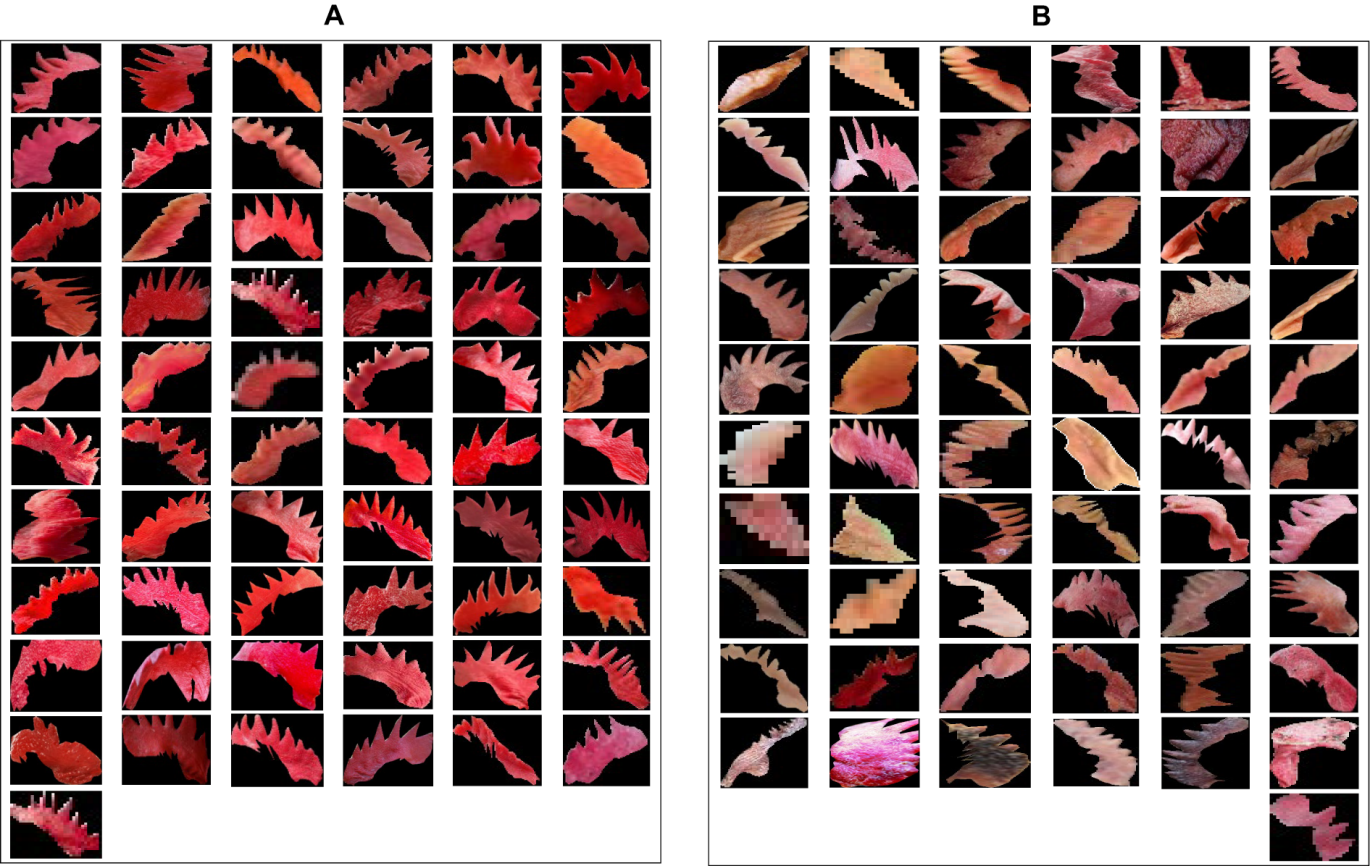
**(A)** Masked images of healthy chicken comb and **(B)** masked images of infected chicken comb.

The difference between healthy and infected chicken comb was illustrated in Figures 4A,B, respectively, using masked images. According to Figure 4, the healthy and infected chicken can be clearly separated based on the chromaticity of the chicken comb, and the impact of infection on the chromaticity value will be further discussed.

**Figure 5:**
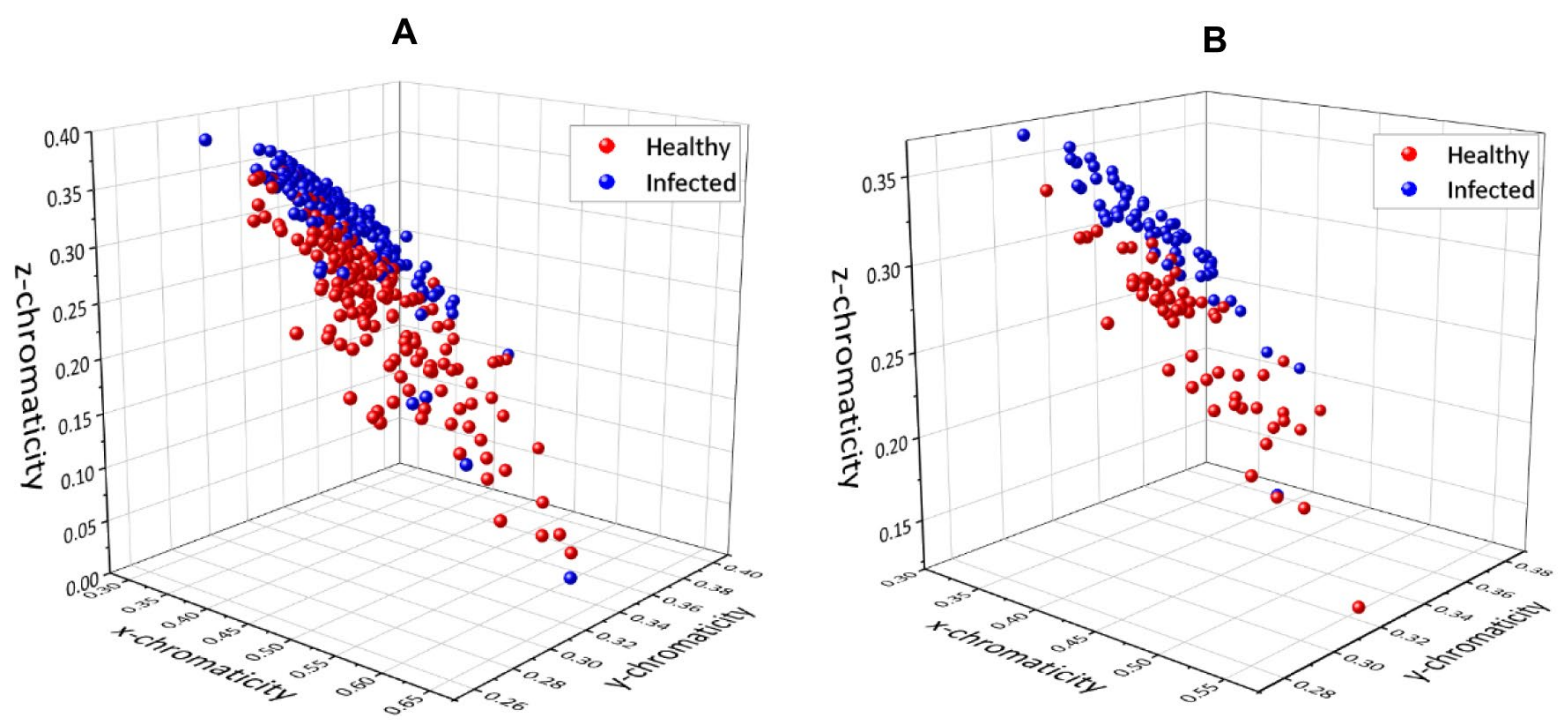
3D plots for chromaticity values *x*, *y,* and *z* using **(A)** pixel-level method and **(B)** pixel-averaging method.

The first set of analyses examines the impact of infection on the three-color space parameter and the correlation between each variable parameter. The 3D scatter plot of *x, y,* and *z* data for the pixel-level method and pixel-averaging method are shown in Figures 5A,B.

**Figure 6:**
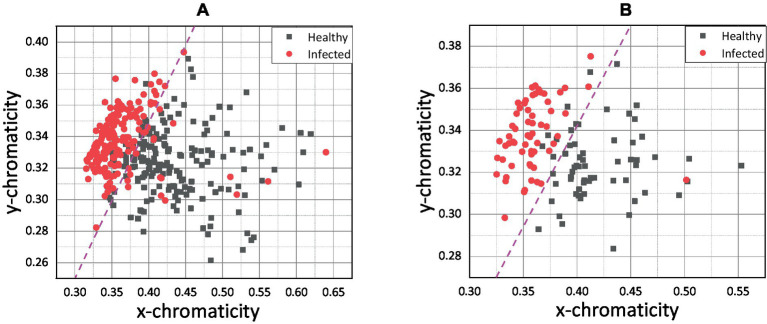
**(A)** *xy* scatter plot for pixel-level method and **(B)** *xy* scatter plot for pixel-averaging method.

The scatter plot of the pixel-level method (Figure 5A) appeared to be more complex because of the total data; three sample points from 61 images resulted in 183 points for each class plotted on the graph. However, Figures 5A,B show that both methods have resulted in the same pattern and no significant difference in the distribution of the scatter plot. It can be seen that the infected and healthy chickens were well separated based on the 3D plot. The results were further analyzed by plotting each component in a 2D plot; *xy, xz,* and *yz.* Figures 6A,B present the chromaticity plot of *xy* chromaticity data for pixel-level and pixel-averaging methods, respectively.

Figure 6 shows that the infected and healthy chickens was well separated by *x* chromaticity for both methods. According to Figures 6A,B, the most infected chicken was scattered below *x =* 0.375, while the healthy chicken was scattered above. The *y* chromaticity value of infected and healthy chickens overlapped, and no specific threshold value can be hypothetically assigned based on the *y* chromaticity variable. However, by combining the *x* and *y* variables, the infected and healthy chickens can be separated more distinctly. Since the scatter plots of healthy and infected chicken were linearly separated, a magenta line was drawn as an indicator line to differentiate between both groups.

Based on the indication line on the pixel-averaging method, it can be observed that only one infected chicken was scattered in the healthy chicken region. On the contrary, 14 infected chickens were spread in the healthy area for the pixel-level method. False classification may occur due to an error in the sampling process. For example, the color of the chicken comb only changes on the front side, and through conventional understanding, the chicken was infected based on that indication. False classification may occur if the sample was taken at the back side of the chicken comb without significant color change. Apart from that, the pixel-averaging method considered all the color data bound in the selected region. Instead of better results in classification, the error and false detection can be reduced. This view was proven by Cao et al. (47), which proposed a new method for water quality detection by considering the average RGB value for the detection (47). Srinivasan et al. (48) also used the average RGB value of each pixel in the image to indicate hemoglobin in human blood for diagnosing anemia.

**Figure 7:**
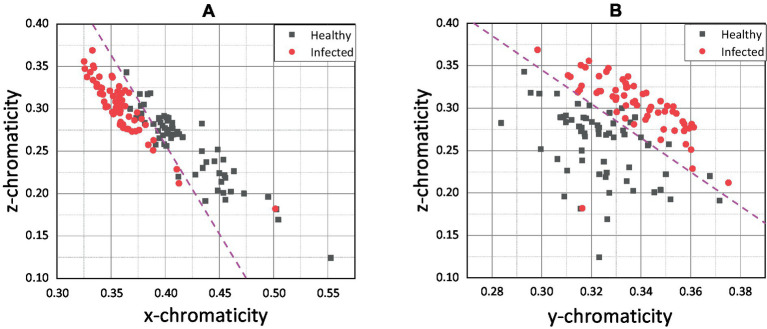
**(A)** *xz* and **(B)** *yz* scatter plot for the pixel-averaging method.

Since the pixel-averaging method was relevant and gave better results in classifying healthy and infected chickens as shown in Figure 6B, further results and discussion on the impact and correlation between the variables and health status will focus on the pixel-averaging method only. Figures 7A,B show the pixel-averaging methods’ results of *xz* and *yz* plots, respectively. Table 1 presents the Logistic Regression’s sigmoid input function (referring to Equation 7) according to the pixel-averaging method dataset.

**Table 1:** Sigmoid input function of the Logistic Regression.

Trained variables/Model	Sigmoid input function, $f\left(x_{1},x_{2}\right)$
xy	f(x,y)=0.29337105+(2.34857535)x+(−0.8202156)y
xz	f(x,z)=0.29103932+(2.65420658)x+(0.37211062)z
yz	f(y,z)=0.26176678+(−1.59957638)y+(−2.22224727)z

The scatter plot of *xz* (Figure 7A) shows that the infected and healthy chickens can be separated based on the threshold value of below *z* = 0.25 for the *z* chromaticity value. When combining the *x* and *z* chromaticity values, the infected and healthy chicken can be separated based on the magenta line as the hypothetical threshold line. Similarly, by combining *y* and *z* (Figure 7B), the infected and healthy chickens can be classified based on the magenta line drawn. Both plots showed that one chicken could be falsely classified as healthy chicken.

The *x* chromaticity variable was the most dominant variable, followed by z and y variables based on the linear regression sigmoid input function results. It can be observed that the *x* chromaticity variable results in a more significant positive classifier coefficient than the *y* variable with 1.5284 higher by referring to the *xy* model (Table 1). The results show the same trend as in the *xz* model when compared with the *z* chromaticity variable, with 2.2821 higher in the classifier coefficient. Therefore, we can conclude that any small change in the *x* chromaticity variable would significantly contribute to the classification of the chicken. Since the classifier coefficient of *x* chromaticity variable results in a positive sign, the increments of *x* value would increase the value of the sigmoid input function; thus, the results of the sigmoid function would converge to 1. Theoretically, the chroma or actual perceived color was indicated by the *x* and *z* values (43). The *x* chromaticity value can be approximately described as green to red part. So, based on our results in Figures 6B, 7A we conclude that the infected chickens were more converging to green because most of the infected chicken points were scattered below healthy chicken in terms of *x* chromaticity value.

Moving on to the *z* chromaticity variable, the classifier coefficient for the *z* variable was 2.2821 lower when referring to the *xz* model. So, any change in the *z* chromaticity value does not significantly contribute to the classifier predicting the chicken’s health. However, according to *yz* model, the *z* chromaticity variable was more dominant than the *y* variable, with 0.6226 higher in the classifier coefficient. Since the coefficient carries a negative sign (*yz* model), increasing the z chromaticity value would encourage the classifier model to predict the chicken to be infected. The *z* chromaticity value can be approximately described as a yellow to blue part for any increment in value (43). Therefore, we conclude that the infected chickens converged more to the blue region according to Figures 7A,B. The weakest variable, *y* chromaticity, has a weaker negative coefficient of 0.8202 compared to the *x* chromaticity variable in the *xy* model. Similarly, in comparison with *z* chromaticity by referring to the *yz* model, the *y* variable resulted in a smaller negative coefficient of 1.5996, while that of the *z* variable was 2.2222. The negative sign indicates that the increased value of y would lower the value of the sigmoid input function; thus, the sigmoid function would converge to 0. The small coefficient of the *y* chromaticity variable was expected based on the *xy* and *yz* plots in Figures 6B, 7B. The scattered point of infected and healthy chicken mostly overlapped in terms of *y* chromaticity value, making the classification nearly impossible. The image data chromaticity’s brightness, luminosity, or lightness were represented by the *y* value (43). According to the results, the *y* value was considered the weakest variable that correlated to chicken health due to no significant difference between healthy and infected chickens, and the classification was nearly impossible. Therefore, a possible explanation for this might be that our data comes from different sources with different illuminants. This finding corroborated with previous research, which found that the redder comb had more excellent cell-mediated immunity or better health condition (35). Moreover, Martínez-Padilla et al. (49) concluded that comb redness or plasma carotenoids were negatively correlated with *Trichostrongylus tenuis* abundance. Plasma carotenoids are pigments responsible for the vivid color red in the chicken comb, while *T. tenuis* is a nematode in birds that cause diseases.

These findings further support the idea of separating chroma and brightness for the detection method proposed in the literature (47), which uses chromaticity values to measure dissolved water content. However, combining the chromaticity value with the brightness makes the classification viable. The present findings were consistent with previous work (41), which considered intensity and chromaticity features in their algorithm to classify daytime and night images. Furthermore, a study on the correlation between comb color and the immunity system of the chicken was performed based on the red chroma, represented by 600–700 nm, relative to brightness (35).

### 3.2. Supervised machine learning results

**Figure 8:**
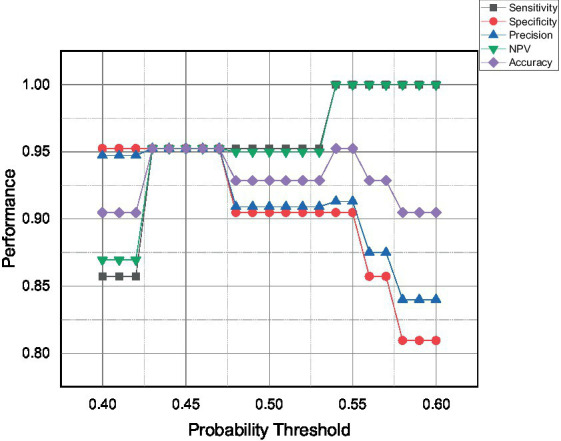
The performance parameter of the Logistic Regression model at varying probability threshold.

This subsection discusses the performance parameter, advantages, disadvantages, and limitations of all the developed classifiers. Since the Logistic Regression model is the only model that can provide a probability value, the current work will iterate the probability threshold from 0.40 to 0.60, and the expected performance of the model is presented in Figure 8.

The model performance can be categorized into three categories; over-, optimum-, and under-predict the positive event or infected chicken. The first category is over-predicted, which can be seen for the probability threshold of more than 0.53. The model starts to over-predict positive events, resulting in the highest possible sensitivity and NPV of 100% with zero false negative events detected. Secondly, the model can be tuned to get optimum performances which can be indicated by a probability threshold ranging from 0.43 to 0.47. The model was expected to predict 95% for all five performance parameters due to the same amount of false positive and false negative events. Lastly, the proposed model was expected to be under-predicted infected chicken when the probability threshold was below 0.43. The present findings seem consistent with other researchers’ views that precision and sensitivity are proportional to actual positive value but have an inverse mutual relationship (50).

Table 2 compares all the supervised machine learning models and notes that the performance of the Logistic Regression was based on an optimum probability threshold of 0.47 and C = 1 for comparison with other models. For SVM models, Linear kernel with C = 1, Polynomial kernel with C = 1 and d = 1, RBF kernel with C = 1 and γ = 0.1, and Sigmoid kernel with C = 1 and γ = 3 were presented. KNN showed the best performance when the K-value was set more than 5, while for the decision tree model, the Gini criterion was better compared to the Entropy.

**Table 2:** Comparative analysis of different types of machine learning algorithms.

Model	Confusion matrix	Performance (%)		Model parameters	Data linearity	Incremental learning	Data fitting	Probability output	Performance tuning	Limitation
Logistic Regression	$\left[\begin{array}{cc} 20 & 1\\ 1 & 20 \end{array}\right]$	Sensitivity	95	C = 1 Threshold =0.47	Linear	Yes	Yes	Yes	Before and after training	Stability of performance during Incremental training (51)
		Specificity	95							
		Precision	95							
		NPV	95							
		Accuracy	95							
SVM-Linear	$\left[\begin{array}{cc} 20 & 1\\ 1 & 20 \end{array}\right]$	Sensitivity	95	C = 1	Linear	Yes	Yes	No	During training	Storage cost from continuous data learning for non-linear SVM (52, 53)
		Specificity	95							
		Precision	95							
		NPV	95							
		Accuracy	95							
SVM-Polynomial	$\left[\begin{array}{cc} 20 & 1\\ 1 & 20 \end{array}\right]$	Sensitivity	95	C = 1 d = 1	Linear/Non-linear	Yes				
		Specificity	95							
		Precision	95							
		NPV	95							
		Accuracy	95							
SVM-RBF	$\left[\begin{array}{cc} 20 & 1\\ 2 & 19 \end{array}\right]$	Sensitivity	95	C = 1 γ = 0.1	Non-linear	Yes				
		Specificity	90							
		Precision	91							
		NPV	95							
		Accuracy	93							
SVM-Sigmoid	$\left[\begin{array}{cc} 16 & 5\\ 2 & 19 \end{array}\right]$	Sensitivity	76	C = 1 γ = 3	Non-linear	Yes				
		Specificity	90							
		Precision	89							
		NPV	79							
		Accuracy	83							
KNN	$\left[\begin{array}{cc} 20 & 1\\ 2 & 19 \end{array}\right]$	Sensitivity	95	k-value = 5	Not applicable	Yes	No	No	Before training	Speed of calculation Data update may deviate (54, 55)
		Specificity	90							
		Precision	91							
		NPV	95							
		Accuracy	93							
Decision tree	$\left[\begin{array}{cc} 18 & 3\\ 1 & 20 \end{array}\right]$	Sensitivity	86	Criterion = Gini	Not applicable	Yes	No	No	Before training	Can cause instability for any data change (56)
		Specificity	95							
		Precision	95							
		NPV	87							
		Accuracy	90							

Logistic Regression, SVM-Linear and Polynomial kernel perform the best in terms of specificity, precision, NPV, and accuracy, followed by SVM-RBF kernel, KNN, Decision Tree, and lastly, SVM-Sigmoidal, in this present study. Logistic Regression, SVM-Linear, and Polynomial kernel perform the best compared to other models because our chromaticity feature data for healthy and infected chicken were linearly separated (Figure 6B). Supporting these statements, researchers in the literature (28) also reported better accuracy using SVM Linear and Polynomial kernel model compared to RBF for their linearly separated dataset.

Incremental learning to extend the model’s knowledge while implementing it in practical applications was possible for all models. However, each model has its advantages and disadvantages during implementation. According to the results, the Logistic Regression, SVM Linear and Polynomial kernels perform the best, with a 95% score for all parameters. Compared with other models, logistic regression can output the results in probability values from 0 to 1, and the classification threshold can be assigned. Thus, the performance of the model can be adjusted. However, instead of tunable performance, the stability of the performance itself was an issue during incremental learning (51). Moving on to SVMs models, the storage cost was a significant drawback for these algorithms due to continuous data learning (52, 53). In addition, kernel selection in developing SVM models is essential as it affects the performance of the model. For instance, the SVM-Sigmoid kernel performed at 76% sensitivity, 90% specificity, 88% precision, 79% NPV, and 83% accuracy, which can be considered the lowest among others.

Next, the KNN model has performed similarly to SVM-RBF. KNN model is much simpler than logistic regression and SVM models because it does not need any data training since its algorithms rely on the number of neighbors or K-value for classification. This model’s primary limitation is the calculation speed during incremental training (54). False detections may also occur when the data becomes more extensive and no change or update makes for the K-value (55). Another non-parametric model, the decision tree, performed with 86% sensitivity, 95% specificity, 95% precision, and 87% NPV, and 90% accuracy. The Decision Tree is easy to train due to no normalization and data scaling are needed. The algorithms for separating the infected and healthy are intuitive and easy to explain. However, the models may become complex due to the number of depths specified in the training process, and any small change may cause significant changes in the tree’s structure (56). Plus, implementing an incremental learning algorithm can variate the stability of the model due to continuous data updates.

In summary, all the models discussed in this subsection can be considered acceptable and successful in classifying health status. Even though current works do not use any specific experimental dataset, all the models have shown to be well developed by just using the randomly well-distributed training and validation image dataset. However, models with high sensitivity, such as Logistic Regression, KNN, SVM-Linear, and SVM-Polynomial, should be considered for current application in providing early warning to prevent major outbreaks. Hicks et al. (50) stated that the consideration of the specificity and precision was based on applications; for medical applications, it is better to over-predict than underestimate the degree of severity. Therefore, current work would consider a model with high sensitivity even though it has a low precision value to over-predict the positive event to prevent significant outbreaks that can cause economic loss and threaten human health.

### 3.3. Comparison with other reported work

The results reported in this work are compared with other related works which predict the chicken health status. Table 3 shows the summary of the related works. Zhuang et al. (28) utilized an SVM-Polynomial model with 99.469% accuracy to classify infected chicken (bird flu) based on all extracted morphological features: concavity, skeleton altitude angle, shape features, linear area rate, elongation, and circularity. Similarly, other works proposed SVM-RBF models with an accuracy of 97.8% based on extracted locomotor features such as circle variance, elongation, convexity, complexity, eccentricity, and mobility features of walk speed (27). These works (27) were compared with the results reported in this work because they used image processing techniques to extract features as predictors to predict infected chicken. Both works extracted all the morphological, locomotor, and mobility features from the chicken images, and the proposed supervised machine learning classifier model’s achieved accuracies >97%. In contrast to these reported works, the results of our work demonstrated that despite only one feature (chicken comb’s chromaticity) being used, prediction accuracy as high as 95% can be achieved. This scenario indicates that the chicken comb chromaticity is a very distinctive feature that can be used to predict the bacteria- or virus-infected chickens, as well as confirming the effectiveness of the machine learning models used in this work. It can also be concluded that high prediction accuracy can be achieved with simpler feature extraction and easier image processing technique, if the accurate and distinctive feature is selected.

**Table 3:** Summary of related works.

References	Features/Input data	Technique	Model/Algorithms	Performance
Zhuang et al. (28)	Concavity, skeleton altitude angle, shape features, linear area rate, elongation, and circularity	Image processing	SVM Polynomial kernel	99.469% accuracy
Okinda et al. (27)	Circle variance, elongation, convexity, complexity, eccentricity, and walk speed	Image processing	SVM RBF kernel	97.800% accuracy
Zhang and Changxi (30)	Abnormal swelling detection	Deep learning	ResNet	95% accuracy 90% sensitivity
Mbelwa et al. (57)	Abnormal dropping	Deep learning	XceptionNet	94% accuracy
Mbelwa et al. (58)	Abnormal dropping	Deep learning	XceptionNet	98.24% accuracy
Zhuang and Zhang (29)	Chicken images, feather texture, posture	Image processing and deep learning	CNN	99.7% precision

This reported work is also compared with the deep learning-based algorithms for detecting infected chicken applications. Zhang and Chen (30) have developed a ResNet algorithm with 94% accuracy to detect infected chickens using abnormal swelling images for their training datasets. Other researchers used different textures of chicken-dropping image datasets to classify healthy and infected chickens using XceptionNet with 94% (57) and 98.24% (58) accuracy after fine-tuned. Compared to our works, both of the works (30, 57) have reported lower accuracy. Similar to our work, these works also utilized only one feature, but our reported work utilized a much simpler image processing technique and lower computational power for training the classifier models. Besides that, Zhuang and Zhang (29) successfully developed algorithms to detect infected chickens with a precision of up to 99.7% by combining image processing and deep learning. To develop these algorithms, authors have utilized the difference in the chicken posture and feather images to train their classifier model. The proposed algorithms were more computationally complex than our work. However, the result performance of the model or classifier was promising. Therefore, it can be proposed that to achieve >99% accuracy, future work will explore on the deep learning algorithms to hybridize with our works to provide early detection algorithms for the prevention of disease outbreaks in poultry farms that can benefit the farmers and improve food safety.

This current work has proven the ability of utilizing the chromaticity of the chicken combs features can be used to detect bacterial- or virus-infected chickens with the help of machine learning models. However, for an implementation in a large-scale chicken farm, a more realistic approach such as capturing the images directly from the chicken cages may be carried out. Further illustration of the accuracy of the model to work in a large -scale poultry farm, by implementing real images dataset, and validation of the model is still needed. Furthermore, hybridization of the chicken comb feature with other established features such as morphological (28), locomotor (27), mobility (27), and optical flow (31), would be future works that need to be considered. The multi-features approach may lead to another breakthrough that would contribute to improved food safety and automation in poultry farm industries.

## 4. Conclusion

This study presents an early prediction algorithm for detecting bacteria- or virus-infected chickens based on the chromaticity of the chicken comb feature. The algorithm extracted the RGB color data at the area of the chicken comb and converted it into the CIE XYZ color space to analyze the effect of bacteria or virus infection on the chromaticity of the chicken combs. The chromaticity data features of healthy and infected chickens were plotted, and the impact of infection on the chromaticity of the chicken comb was analyzed. Machine learning methods were used to predict the chicken’s health status based on the chromaticity feature. The performance analysis of the developed machine learning models proved that the classification of healthy and infected chicken is viable based on the chromaticity of the chicken comb features. All the developed models have excellent generalization to recognize the infected chicken. The results suggest that the chicken comb chromaticity-based algorithm can provide prediction and detection of infected chicken. This algorithm can be applied as a disease monitoring system for the chicken on the farm. In addition, this algorithm can be integrated with other morphological, locomotor, and mobility-based algorithms for detecting infected chickens. Thus, the risk of significant diseases outbreak on the farm could be minimized.

## Data availability statement

The datasets presented in this study can be found in online repositories. The names of the repository/repositories and accession number(s) can be at: https://github.com/anifakhmal/Infected-vs-Healthy-chick.git.

## Author contributions

MAB: methodology, data curation, and writing-original draft preparation. PK: conceptualization, formal analysis, supervision, project administration, writing-review, and editing. ST and AO: supervision, validation, writing-review, and editing. MZB: software, validation, writing-review, and editing. HL: visualization, writing-review, and editing. All authors have read and approved the final manuscript.

## Funding

This work was supported by the Ministry of Higher Education, Malaysia, through the Fundamental Research Grant Scheme (FRGS), under the project code of FRGS/1/2020/TK0/UNITEN/02/6 (20210111FRGS). MAB would like to express his gratitude toward the financial support provided by the project under the graduate research assistant scheme. The article processing charge was supported by Tenaga Nasional Berhad (TNB) and UNITEN through the BOLD Refresh Publication Fund under the project code of J510050002-IC-6 BOLDREFRESH2025-Centre of Excellence.

## Conflict of interest

The authors declare that the research was conducted in the absence of any commercial or financial relationships that could be construed as a potential conflict of interest.

## Publisher’s note

All claims expressed in this article are solely those of the authors and do not necessarily represent those of their affiliated organizations, or those of the publisher, the editors and the reviewers. Any product that may be evaluated in this article, or claim that may be made by its manufacturer, is not guaranteed or endorsed by the publisher.

## Footnotes

^1^https://github.com/anifakhmal/Infected-vs-Healthy-chick.git
